# Supplementary material for: Innovation of heterochromatin functions drives rapid evolution of essential ZAD-ZNF genes in Drosophila
Source: eLife. 2020 Nov 10;9:e63368. doi: 10.7554/eLife.63368 (PMC7655104; doi:10.7554/eLife.63368)
Supplement: Figure 3—source data 1. — Total number of flies counted across all replicates are shown. Numbers in parentheses refer to expected numbers in case of full rescue. The Nnk-mel rescue allele can rescue otherwise inviable piggyBac-null/piggyBac-null, CRISPR-null/CRISPR-null, and NnkRNAi/Act5C-Gal4 progeny. [file elife-63368-fig3-data1.docx]

| **Genotype** | **Rescue with transgene** | **Rescue** | **Heterozygous** | **Total** | **Replicates** |
| --- | --- | --- | --- | --- | --- |
| *piggyBac* null  *piggyBac* null | yes | 64 (71) | 221 (214) | 285 | 3 |
| CRISPR null  CRISPR null | yes | 46 (44) | 130 (132) | 176 | 3 |
| Nnk^RNAi^ Act5C | yes | 169 (161) | 313 (321) | 482 | 9 |

**Figure 3 source data 1**
