# Supplementary material for: Innovation of heterochromatin functions drives rapid evolution of essential ZAD-ZNF genes in Drosophila
Source: eLife. 2020 Nov 10;9:e63368. doi: 10.7554/eLife.63368 (PMC7655104; doi:10.7554/eLife.63368)
Supplement: Supplementary file 2. [file elife-63368-supp2.doc]

>CG17803-ana

RCRICYRRDSNDKDLCADENNVLLYYIEIIAGVRIKKEEGMPDRICSNCHESLQRAMEFRSNCLKTDREKHRTKPLSYGCDVCGHAFRLSCHLEKHMLRHNNTKNFQCPECPRKFYVSYDCNIHIRVRHRGEKPFTCNHCSESFPSGGLRQKHERNAGPRRLPFHCEICLKGFLLECQLKDHMLVH-TGKRSFWCELCDVYYRYKYNLTKHESSSLHLGN

>CG17803-kik

KCRTCSQLDDTVKDLYDKRNIELLHQIKSVTGIWIKAGQGLPHHICESCQEALKKAIEFRDNCIQANRLKHLAKPFSYVCDKCGHAFRMPDQLQIHLLRHNQTKNFACPECPKKFYNSYLRNMHQRVWHRGEKPWSCNHCSETFSNANCRQRHEKEVGPRKLPFHCEICLKGFLLRCQLKEHQVVH-TGERPYWCEICDIYYRFRRNLDKHNKSDAHKNK

>CG17803-mel

KCRTCFRRHEDAQDLYDRVNIALLHHIKVITGVWIQQGKELPHHICSTCQETVNKSMEFRAKCQQVDRERIQAKPLNYVCDKCGHTFRQRSQLQMHLLRHNRAKNFECPECPKKFYDLYTRNIHVRALHKGEHPFPCNHCNESFANASSRHRHERDAGNRKFPIRCDICLKGFLLRSQLTKHQDVH-TGMHPHRCEICDVHYRHRYNLNKHKNTDLHRDN

>CG17803-sec_

QCRTCFRRHEDAQDLYDRVNMALLHNIKVITGVWIQPGKELPHHICATCQETLNKSVEFRAKCQQVDRERVQAKPLNYVCDKCGHSFRQRSQLEMHLLRHNRAKNFECPECPKKFYDLYTRNIHVRAMHKGEHPFPCNHCNEAFANASSRHRHERDAGNRKFPIRCDICQKGFLLRSQLTKHQDVH-TGMRPYRCEICDVHYRHRYNLNKHKTTDLHRDN

>CG17803-sim

QCRTCFRRHEDAQDLYDRVNMALLHNIKVITGVWIQQGKELPHHICASCQETLNKSVEFRAKCQQVDRERIQAKPLNYVCDKCGHSFRQRSQLEMHLLRHNRAKNFECPECPKKFYDLYTRNIHVRAMHKGEHPFPCNHCNESFANASSRHRHERDAGNRKFPIRCDICQKGFLLRSQLIKHQDVH-TGMRPYRCEVCDVHYRHRYNLNKHKTTDLHRDN

>CG17803-ere

KCRTCFRRDGGVKDLYDRDNMALLHHIKVATGVWIQQGKELPHHICATCQETLKKTVEFRAKCQQIDRERMQAKPLNYVCDKCGHSFRQRSQLQMHLLRHNSAKNFECPECPKKFYDLYTRNIHIRALHKGEHPFSCNHCNESFSNASSRHRHEKDAANRKFPFRCDICLKGFLLRSQLIKHQDVH-TGKRPHRCELCDVHYRHRYNLNKHKSSILHRDN

>CG17803-yak_

KCRTCFRRDEGVKDLYDRDNMALLHHIKVTTGVWIQQGKELPHHICATCQETLKKAVEFRGKCQQIDRERVQAKPFNYVCDKCGHSFRQRIQLQMHLLRHNSAKDFECPECPKKFYDVYTRNIHVRVRHKGEHPFRCNHCNESFSNASSRHRHERNAGNRKFPFRCDICLKGFLLRSLLTKHLDVH-TGKRPHRCELCDVHYRHRYNLNKHKTSKLHRDN

>CG17803-tak

KCRTCFQLDEDVKDLYERDNIALLYHIEVTTGVWIETGEGLPHHICATCQETLQKSVDFRDKCIQIDRERVRAKPFSHICDKCGHSFREQGQLQMHLLRHNKVKNFDCPECPKKFYDAYTRNIHLRARHKGETPFSCNHCNQSFPNASSRHRHEQKAGSRKFPFYCDICSRGFLLRSQLIKHQDVH-TGDRPHWCDFCDVHYRYKYKLNEHYNSNMHKNS

>CG17803-bia

KCRTCFQLDGDVKDLYDRDNIALIYHIEVTTGVWIETGKGLPHHICATCQETLQKSVDFRDKCIQIDRERVRAKPFNYVCDKCGHSFRQPGQLQMHLLRHSSAKNFDCPECPKKFFDAYTRNIHLRARHKGEKPFPCNHCNKSFPNASSRHRHERKAGSRKFPFHCDICFKGFLLRSQLITHRDVH-TADRPHWCEFCDVHYRHRYKLNIHYKSSLHKNN

>CG17803-suz

KCRTCFQSDEDVKDLYERDNIALLYHIEVTTGVWIETGKGLPHHICATCQETLQKSVDFRDKCIQIDRERVRAKPFNHVCDQCGHSFRQRGQLRMHLLRHNSAKNFDCPECPKKFYDAYTRNIHLRARHKGENPFPCNHCNTSFSNASSRHRHEREVGSRKFPFHCDICSKGFLLRSQLINHRDVH-TADRPHWCEFCDVHYRHRYKLNIHYNSSMHKNN

>CG17803-fic

KCRTCFRLDGNVKDLYAKDNVDLLYRIKLTTGVRIQSGKGVPHHICATCQNTLKTAIEFREKCIRIDREWAREKPLNHICQKCGHSFRHPGQLQMHMLRHDRAKNFECPECPKRFYDAYTRNMHIRVLHKGENPFPCNHCNMSFSSGSSRHKHEVKAGPRKFPYHCDICSKGFLLRCKLTEHQLVH-TAERPHWCEFCDVHYRQKYNLNKHYNSNMHKNN

>CG17803-ele

KCRTCFELDTNVKDLYDKKNSGLRYDLESLTGVWIQRGKSLPHHICKTCQDDLKKSIEFRDKCIQIDRERVQAQPFKHVCEQCGHSFRQVCHLQMHLLRHNSSKNFDCPECPKKFYDSYSRNIHLKVRHKGENPFPCNHCSESFASGSTRHKHEREAGPRKFPFYCEFCLKGFLLRCKLNEHQKIH-TGERPYCCEICDAYYRYRYNLNKHYKTKTHHNN

>CG17803-rho

KCRTCLRLNTNVKDLYDKDNVGLRYDIESLTGVWIQRGQILPHHICETCQDALKRSIAFRDKCIQIDRERVQAKPLNHVCDKCGHSFRQSGQLQMHLLRHNSTKNFDCPECPKKFYDSYSRNIHLKVRHKGVKPFPCNHCNQSFASGSGRHRHEREAENRKFPYHCEFCLRGFLLRCKLAEHQLVH-TGERPYCCEFCDVHYRHRYNLTKHYNSNMHKNN

>CG17801-kik

QCRLCVRYELEPIVLF---NHEVLSKIKDITGLTLDDEEHLPRHICAICQKQLTLASGFRDRCRRVQPR--PKAREFLICDQCGHSFKNSTNLKMHKLRHSGLKDFACTSCDQQFVNPYLLKVHIRVRHLGERPFACRFCERKFFTSGARTYHQRTQHIRDGSYRCDLCSNVFNTKSDLNSHKYSH---RKPFQCEVCNVSFSRRCNLKHHYASKRHCKI

>CG17801-ana

KCRLCGKLAWQPKNLF---NREIIARIKVITGLYLREEKHLPRHICTRCYDQLQLSTGFRERCLRNQRAKAPRKDLELQCNMCGKYLKTVDNLKNHRLRHLGVKNFACEICNKRFVTKHLLTLHERVRHLGERPYPCGYCQLCFFTSSARNCHERLQHIRDKRYQCDECGKQFNTPTFLKRHLFAH-TGQKPHSCETCSVSFSRTEYLKNHLRSKKHQKM

>CG17801-fic

DCVVCI-SATRAPRFYS--KAEFSSRSKDCRNLQLYEMEDLPRHICPSCLKDLNISFKLRKQIIWTHKRRRNRKVTSLECTECGKCLKSTENLKMHILRHKGQKDFSCSYCDRQFVSKHLLNLHNRVRHLGEQPFKCTFCPSTFFTSSSKSRHEQVKHIRSRNFKCDECSSEFKTKSCLNKHKIIH-TGKKPYLCDLCNKKFSRREKLTSHFKSQTHQKR

>CG17801-ele

QCRLCCHRQENPMIIF---NRDVLAKVKDLTGLSLREEKHLPRHICPSCLKDLNTSIKLRDRIGRNHRKMKTRKTRSRECTECGKVLKSLNNLKLHMLRHTGKKDFICNLCDRRFVSIHLLNLHKRVRHLGEQPFKCRFCQKSFSTSTAKSRHEVMKHVRDGSYMCDECGRQFHTQTCLNNHKVLH-AGNKPFYCDICHIYFSRRKSMKRHFQSQAHQKK

>CG17801-rho

QCRLCGLRHKNPRIIF---NRGVLAKVKAVTGLSLREEEHLPRHICPSCLKELNMTITLRKRIRQNHKRIGTRKTQSLQCTKCGKLFKTNYNLKMHLLRHTGQRDFACNFCDRRFVSIHLQKLHQRVRHMGERPFKCSFCHDTFFTSTAKNRHEQMKHIRDRRYICDECGKQFRTKTCLNHHKILH-TDMNAFYCDICNVNFSRKKNMKRHFQSQAHQKR

>CG17801-mel_

QCHLCASCHLNPTIIF---CLEVLAKIKDLTGIWLEQNERQPRHICPSCLNDLNTSIKLKKRIQRVHKR-TARKMQSLVCPKCGRVFKTPYNLKTHMVRHTGEKNFPCTFCDKRFVTKYLARLHERVRHMGEQPFECNFCSATFFTSTAKSSHERIRHIRDLRYQCDQCTKRFNTKTCLNKHKFLH-SGLKPFDCVICQINFARKATLRSHFDSVAHQKR

>CG17801-sec_

QCHLCASRHLNPTIIF---SLEVLAKIQDLTGIWLEQNERQPCHICPSCLNDLNTSIKLKNRIQRVHANRRARKIQSLECPKCERVFKTPYNLKTHMARHTGEKNFPCTFCDKRFVTKYLVRLHERVRHMGEQPFKCNFCSETFFTSSAKSRHERIRHIRDLSYQCDKCTKRFNTKTCLNKHKFLH-TGLKPFDCVICQINFARKAALRRHFDSVAHLMR

>CG17801-sim

QCHLCASRRLNPTIIF---SLEVLAKIKDLTGIWLEQNERQPRHICPSCLNDLNTSIKLKNRIQRVHTKRRARKIQSLECPKCERVFKTPYNLKTHMVRHTGEKNFPCTFCDKRFVTKYLVRLHERVRHMGEQPFKCNFCSKTFFTSSAKSRHERIRHIRDLSYQCDQCTKRFNTKTCLNKHKFLH-TGLKPFDCVICQINFARRAALRRHFDSVAHLIR

>CG17801-ere

QCQLCGLFHLNPTTIF---SLRVLAKIKDLSGLWLEQDDQQPRHICPTCLNDLNTSIKLKRRIQRIHKR-RTRKMQSLECPECRMVFKTAYNLKIHLVRHTGQKDFQCAFCDRRFVSKYLGRLHERVRHMGEQPFSCQFCSDTFFTSSAKSRHERMRHIRDQSYQCDECGKRFNTKTCLNKHKFLH-TGLKPFSCDICNIDFAQKSKLRNHFNSQAHQKK

>CG17801-yak

RCQLCDSCHLSPTSIF---SLGVLAKIKDLTGLWLEQEDHQPRHICPSCLNDLNTSIKLKRRIQRIHKR-GTRKMQSLECPECRRVFKTAYNLKIHLVRHTGQRDFQCTFCERQFVSKYLWRLHERVRHMGEQPFRCKFCPDTFFTSSAKSRHERTRHIRDRSYQCDECSKRFNTKTCLNKHKFLH-TGLKPFGCDICHIDFARKSKLRNHFNSLAHQKK

>CG17801-eug

QCRLCGLCHQNPMSIF---SSDVLSKLKDLTGLELHEEEHQPRHICSTCLNELKVSIKFKERILRSRKK-RARKTQSLDCKECGKSFKSTYNLRIHLVRHTGKKDFACIFCDRRFVSKLLLKLHVRVRHLGEQPFKCKFCPNTFFTSTAKYLHERRRHIRDWKYQCDECFKRFNTKTSLNKHKFLH-TGKRPYCCDICNINFAERYTLKCHFKTKSHYNK

>CG17801-bia

QCRLCALFQLNPTIIF---NRGILAKIKDLTGLWIQEDEHLPRHICPSCLNELSIFTELRQRIQRNHKRKRTRKAQCLDCTECGKSFKTTYNLKLHLVRHTGRKDFACSFCERQFVSKHLLKLHERVRHLGEQPFKCKFCPNTFFTSTAKNQHERIRHIRDRSYKCDECNRGFNTKTCLNKHKFLH-TGIKPYYCAVCNINFAEREKLKKHISTQNHQKR

>CG17801-suz

QCRLCALFQLKPTIIF---NSGILAKIKDLTGLWLQEEEHLPRHICPSCLNELSIFTELRQRIQRNHKRRRACKALSLDCTECGKSFKTTYNLRLHMVRHTGQKDFACSFCERQFVSIHLKKLHERVRHLGEQPFKCKFCPDTFFTSTAKNRHERIRHIRDGSYKCDECNSKFNTKTCLNKHKFLH-TGKKPYCCDICNISFAERQKLKNHNQTQIHQKR

>Odj-wil

KCRACADVTANPKDIFTNENKAVLKNIRSLTGLEFNYSEDLPTHICTCCYLDLQHAALFRERCIKAQRKPYYTGDKKYTCEHCGWSFRDCGNMLAHRRTH-GEPTFQCDECARKFYTQPLLNLHIRVRHRNELPFICKYCGQGFPNSPARCRHERTMHPNELPFRCSICPKTFISKISLDKHIQFHKDGEKTHICTSCNKQFVDKQTLKKHYETKYHRRR

>Odj-per

ECRVCGEFISDPKNIFDKENSEILLRIKQLTGLALMFSENLPMHICSCCLLDLNQAVVFRDRCLRTQRKRIPCPEKKYVCDQCGWAFNDLSNMKDHKLRHSE-KQFACDECGSYFYTSRQLKMHVRVKHKGEKPFLCKYCGMAFNNSPSRCRHERRYHSNDLPYVCNLCSKRFVSKVGLTKHALLH-KGGGNHYCEICDKEFKEAIFLRGHYLTKYHRTR

>Odj-pse

ECRVCGEFISDPKNIFDKENSEILLRIKQLTGLALMFSENLPMHICSCCLLDLNQAVVFRDRCLRTQRKRIPCPEKKYVCDQCGWAFNDLSNMKDHKLRHSE-KQFACDECGSYFYTSRQLKMHVRVKHKGEKPFLCKYCGMAFNNSPSRCRHERRYHSNDLPYVCNLCSKRFVSKVGLTKHALLH-KGGGNHYCEICDKEFKEAIFLRGHYLTKYHRTR

>Odj-ana

ECRICGEFTADPKNIFERRNRRILIAIKQVTGLALKFEAQLPMHICSCCLLDLSHAIAFRARCLQTDRR-PNLNDKRYVCDQCGWSFADLSNMKDHKLRHYDEK-FVCNECGRKFYTQPTLKMHIRVIHNGEKPYVCQYCGEGFGNSPARCRHERIYHYDELMFPCDYCDKRFNSDKGRMKHQIKC-NAEETFYCEPCDKEFKTADCLRRHHFSKYHRKR

>Odj-kik

ECRLCCGFTPHPKNLFEKRNQRILTAIQQITGLEIVLETTLPKHICANCLLDLSHAVAFRQRCLQAHRPKPYPKDKRYVCDQCGWAFADMSNMKDHKLRHFE-KKYACDQCGRKFYTQPLLRLHFRVHHMGEKPYVCKYCGMGFNNSPARCRHERQVHPNELAFKCKICNKRFNSEKSRTKHQDGH-KSNEPHYCETCNKEFKEASFLHRHFATKYHRKR

>Odj-eug

ECRICAEFTPNPKNIFEKRNQPIRTAIEQITGLEIVLEKLLPQHICSCCLLDLSQALAFRQRCLQTHKK-VDRSIKRYVCDQCGWSFNDHSNMKDHKLRHFAEK-FSCEECERKFYTMPLLRLHVRVHHRGEKPYVCKFCGMGFGNSPSRCRHERQMHPNELCFSCRICGKRFNSEKGRSKHEEGH-KSDQPHLCLTCNKEFKEAQFLQRHYSTKYHRKR

>Odj-fic

ECRICAEFTPNPKNIFEKKNHRILTAIEQITGLEVVLEELLPQHICSCCLLDLSHAVNFRQRCLKTHPK-VDRSIKRYVCDQCGWSFNDMSNMKDHKLRHFEEQ-FSCDECGRKFYTMPLLRLHIRVHHKGEKPYVCKFCGMGFANSPSRCRHERHVHPNELSFPCGICGKRFNSDKGRNKHEEGH-KSNKPHICLTCNKEFKEAQFLTRHYTTKYHRKR

>Odj-ele

ECRICGEFTPHPKNIFEKRNHRIRTAIEQITGLEIVLEKLLPQHICSCCLLDLSHAIAFRQRCLQTHPK-VDRSIKRYVCDQCGWSFNDHSNMKDHKLRHFEEQ-YSCDECGRKFYTKSLLRLHDRVHHKGEKPYVCKFCGMGFANSPSRCRHERQMHPNELSFPCGVCGKRFNSDKGRNKHQEGH-KNNQPHICLTCNKEFKEAQFLNRHYSTKYHRKR

>Odj-rho

ECRICGEFTPHPKNIFEKRNHRIRTAIEQITGLEIVLEKQLPQHICSCCLLDLSHAIAFRQRCLKTHPK-VDRSIKRYVCDQCGWSFNDMSNMKDHKLRHFEEQ-FSCDDCGRKFYTMPLLRLHIRVHHKGEKPYICKFCGMGFANSPSRCRHERQVHPNELSFPCGICGKRFNSDKGRNKHQEGH-KSNRPHICLTCNKEFKEAQFLHRHYSTKYHRKR

>Odj-ere

ECRICGEFTPHPKNIFEKRNHRIRMAIEQITGLEIVLEKMLPQHICSCCLLDLTQAVAFRQRCLQTHPK-VDRSIKRYVCDQCGWSFNDHSNMKDHKLRHFEEK-FSCDECGRKFYTMPLLRLHIRVHHKGEKPYVCKFCGMGFANSPSRCRHERQMHANELVHPCKICGKRFNSEKGRLKHEEGH-KSDQPHICLTCNKEFKEAQFLHRHYATKYHRKR

>Odj-yak_

ECRICGEFTPHPKNIFEKRNHRIRMAIEQITGLEIVLEKMLPQHICSCCLLDLTQAVAFRQRCLETHPK-VDRSIKRYVCDQCGWSFNDHSNMKDHKLRHFEEK-FSCDECGRKFYTMPLLRLHIRVHHKGEKPYVCKFCGMGFANSPSRCRHERQMHANELVHPCKICGKRFNSEKGRLKHEEGH-KSDQPHICLTCNKEFKEAQFLHRHYSTKYHRKR

>Odj-mel_

ECRICGEFTPHPKNIFEKRNHRIRMAIEQITGLEIVLENMLPQHICACCLLDLTQAVAFRQRCLETHPK-VDRSIKRYVCDQCGWSFNDHSNMKDHKLRHFEEK-FSCDECGRKFYTMPLLRLHIRVHHKGEKPYVCKFCGMGFANSPSRCRHERQMHANELVHPCKICGKRFNSEKGRLKHEEGH-KSDQPHICLTCNKEFKEAQFLHRHYSTKYHRKR

>Odj-sec_

ECRICGEFTPHPKNIFEKRNHRIRMAIEQITGLEIVLEKMLPQHICACCLLDLSQAVAFRQRCLDTDPK-VDRSIKRYVCDQCGWSFNDHSNMKDHKLRHFEEK-FSCDECGRKFYTMPLLRLHIRVHHKGEKPYVCKFCGMGFANSPSRCRHERQMHANELVHPCKICGKRFNSEKGRQKHEDGH-KSDQPHICLTCNKEFKEAQFLHRHYSTKYHRKR

>Odj-sim

ECRICGEFTPHPKNIFEKRNHRIRMAIEQITGLEIVLEKMLPQHICACCLLDLSQAVAFRQRCLDTHPK-VDRSIKRYVCDQCGWSFNDHSNMKDHKLRHFEEK-FSCDECGRKFYTMPLLRLHIRVHHKGEKPYVCKFCGMGFANSPSRCRHERQMHANELVHPCKICGKRFNSEKGRQKHEEGH-KSDQPHICLTCNKEFKEAQFLQRHYSTKYHRKR

>Odj-tak

QCRSCGEFTPQPKNIFEKRNHRIRTAIEQITGLEIRLEALLPQHICSCCLLDLSQAIAFRQRCLKTHPK-VDRSIKRYVCDQCGWSFNDHSNMKDHKLRHFAEK-YSCDDCGRKFYTLPLLRLHVRVHHKGEKPYVCKFCGMGFANSPSRCRHERQVHPNELTHACRICQKRFNSEKGRAKHEEGH-KSDQPFLCLTCNKEFKEGQFLQRHYTTKYHRKR

>Odj-bia

ECRICGEFTPHPKNIFEQRNHRIRTAIEQITGLEIVLEKLLPQHICSCCLLDLSQAIAFRQRCLKTHPK-VDRSIKRYVCDQCGWSFNDHSNMKDHKLRHFAEK-FSCDDCGRKFYTMPLLRLHIRVHHKGERPYVCKFCGMGFANSPTRCRHERLVHADQLAHACKICGKRFNSDKGRAKHEEGH-KSDQPFFCVTCNKEFKENSFLQRHYTTKYHRKR

>Odj-suz

ECRICGEFTPHPKNIFEKRNHRIRTAIEQITGLEIVLEKLLPQHICSCCLLDLSQAIAFRQRCLKTHPK-VDRSIKRYVCDQCGWSFNDHSNMKDHKLRHFAEK-FSCDDCGRKFYTMPLLRLHIRVHHKGERPYVCKFCGMGFANSPTRCRHERQVHADQLVHACKICGKRFNSEKGCAKHEEGH-KSDQPYFCVTCNKEFKENSFLQRHYTTKYHRKR

>Nnk-kik

QCRTCGQYSNNAINIFEG-NDVIINQIALVTGVKLTDKLDFPKRMCFSCLVSLNEAIQFRELCVATNERKKYRARKIYFCDQCGKEFNDKGNLNLHLVRHTGVKPFECPDCGKREFSMYLMNIHIRVKHRGEKPFVCKYCDESFSDSTQRSRHQSRLKVANRRYNCAFCDLRFEVQSQLKKHEIVH-SGQRNYPCEICNVAFTRKFNLQTHFRSRQHKKN

>Nnk-fic

HCRSCGKYSQNPLNIFEG-TRKIVKKFALLTGIWLTDQLYLPKNICACCHVSLNRAIAFRELCIKTNQQGKSKGAKIYFCDQCGRQFNDKGNLNLHLVRHSGVRPFQCPECDRREFSTYLLKIHIRVKHRGEKPFACKFCDERFVNSTKRTRHQTRNKVINKTHTCLHCGICFESKSHLKIHEVVH-SGERNFHCEICNMSFTRSFNLKTHFRSKQHRKK

>Nnk-mel_

QCRLCGDYTQNPVNIFDE-NSKMVRQIALVTGLWLTDHSKMPRNMCSCCLLSLKSAIAFRQACIKTNKRKTYISQKVHICDHCGKKFTDKGNFNLHVLRHSGVKPFECPECGQKEFNRYILNIHIRVKHRGEKPYACQFCDERFVHSTMRSRHENRNKKTPKNFKCNYCDKRYESNYQRAKHEVVH-TGERNFHCEVCKVSFTRNSNLKTHYRSRQHQNK

>Nnk-sec_

QCRLCGDYTQNPVNIFDE-RSNMVRQIALVTGLWLTDHSKMPRNMCSCCLLSLKSGIAFRQVCINTNKRKTYISRKVHVCDHCGKKFTDKGNFNLHVLRHSGVKPFECPECGQKEFNRYILNIHIRVKHRGEKPYACQFCDEKFVHSTMRSRHENRNRKSPKNFKCNYCDKRFESNYQRAKHEVVH-TGERKFHCEVCKVSFTRDSNLRTHYRSGPQQRK

>Nnk-sim

QCRLCGDYTQNPFNIFDE-RSNMVRQIALVTGLWLTDHSKMPRNMCSCCLLSLKSAIAFRQVCINTNKRKTYISRKVHVCDHCGKKFTDKGNFNLHVLRHSGVKPFECPECGQKEFNRYILNIHIRVKHRGEKPYACQFCDEKFVHSTMRSRHENRNRKSPKNFKCNYCDKRFESNYQRAKHEVVH-TGERKFHCEVCKVSFTRNSNLRTHYRSGQHQRR

>Nnk-ere

QCRLCGDYTQNPVSIFDK-RSKTVRQIALVTGVWLTDHSKLPRNMCSCCLLSLKSAIAFRHVCIKTNKRKTYVSHKVHVCDHCGKKFTDKGNFNLHVLRHSGVKPFECPECGQKEFNRYILNIHIRVKHRGEKPYACQFCDERFVHSTMRSRHENRNRTTPKNFKCNYCDKRFESNYQRAKHEVVH-TGERNFHCELCKVSFTRKSNLKTHYRSRLHQKK

>Nnk-yak_

QCRLCGDYTQNPVSIFDK-RSKTVRQIALVTGVWLTNHLKLPRYMCSCCLLSLKSAIAFRHVCIKTNKRKTYVSRKVHVCDHCGKKFTDKGNFNLHVLRHSGVKPFECPECGQKEFNRYILNIHIRVKHRGEKPYACQFCGERFVHSTMRSRHENRNRATPKNFKCNYCDKRFESNYQRSKHEVVH-TGERNFHCEVCKVSFTRNSNLKTHYRSRLHQKK

>Nnk-eug

QCRSCGDYTKNSVNIFEG-SSDIIKQVTLLTGIVLTDQHKLPRNMCACCVVSLKSAIAFRELCIRTHKRKRYICNKTYVCDQCGRKFNDKGNLNLHVLRHTGVRPFKCPECDKKEFNMYLLNIHIRVKHRGEKPFACKYCGECFVNSTKRSRHQLRHKVKPKPLKCHSCDLRFEILSQLKKHEVVH-TGVRNFSCKLCDMSFSRNSNLKTHFKSKQHMKM

>Nnk-tak-2_1

QCRLCGDYTKDPVDIFKG-SSELTKHIALLTGISLSDHFDLPRNMCECCLLSLKSAIAFRELCIKTNKRKRYKSTKTYVCDHCGRKFNDKGNLNLHVLRHTGVRPFECPECGQKEFNKYILNIHIRVKHRGEKPYACKYCDEKFETSIKRTRHSRKHQIKPKPYKCSACDKRFELPSLLTKHEQVH-SGERKFSCDLCHKSFTRSGNLKTHFKSMQHKNN

>Nnk-bia-1

QCRLCGDYTKGALNIFNG-SKQIIVQIALLTGIWLKDQPNMPRKMCSCCLLSLKSAIAFREVCIKTNNRKKYKSSKVFVCDHCGRKFNDKGNLNLHVLRHTGVKPFECPECGQREFNKYILNIHIRVKHRGEKPYACKYCNAQFESSTKRTRHERRQPVKQKPFKCSSCDLRFEIRSQRTKHEKIH-SGIREFQCKVCKVAFTRASNLKIHCKSKQHQRK

>Nnk-suz

QCRLCLDYTKNPVNIFNG-SKEIIVQIALLTGIWLKDQGNLPRNMCSCCLLSLKSALAFREVCIKTNKRKKYKSTKIYVCDHCGKEFTDKGNLNLHVLRHTGIKPFACPECGKREFNKYSLNIHIRVKHRGEKPYACKYCELHFESSTKRTRHERRQTVKQKPFKCSSCDLRFEIRSQRTKHEKVH-SGEREFQCKVCKVAFIRASNLKTHFKSKLHQRK

>Nnk-per

RCRTCGEYSLNATNLFEG-DNDIVRKISFLTGIWITDQLDMPRNICQICQMDLNTAMLFRERCIRTHRKRQNRVNQNYICDQCGKHFNDKGNLNLHLVRHTGVRKFQCPECSHKEYSQFLLKMHIRVKHRREAPYACKYCDERFVCSNKRYRHQRTLKTTDKPYACNFCNHRCNSMSNLRKHEVVH-SGDRPFRCEICDVAFYRMSNLRTHFRSKTHKKK

>Nnk-pse

RCRTCGEYSLNATNLFEG-DNDIVRKISFLTGIWITDQLDMPRNICQICQMDLNTAMLFRERCIRTHRKRQNRVNQNYICDQCGKHFNDKGNLNLHLVRHTGVRKFQCPECSHKEYSQFLLKMHIRVKHRREAPYACKYCDERFVCSNKRYRHQRTLKTTDKPYACNFCNHRCNSMSNLRKHEVVH-SGDRPFRCEICDVAFYRMSNLRTHFRSKTHKKK

>Nnk-tak-2_2

LCRTCGQEAEHAKTLFDREGSNVLCNILKLTGIWFGNHPGVPTKICFSCVLDLNSAVAFRERCIKTNKRKRKKSATIYVCDHCGKQMNDKGNLDTHVLRHTGVRPFECPECDHREINRYTLNIHIRVKHRGEKPYACKYCDEKFETSIKRTRHTRRKLLELKPYKCSFCGKGFVLNYERTSHEKLH-YAEREFTCEICDMSFNRSHNLNLHFKSKNHKRK

>CG17806-tak-3

KCRICGLLGNFGRKLFDIDGEGHLKNIFSLTGIWLIDKPGVPSTMCLSCLLDLNAAVAFRQRLIRTNRPRKVQQVCFFSCEECGTSFMEKDEYEDHLLRHSSFKLFQCEECNHQENTAHRLNLHVRIKHRGELPYMCRFCGQSFDRIMKKLRHER-------------CHKEILTPRAFKNH------------CXXXXXXXXXXXXXXXXXXXXXXXXX

>CG17806-eug

TCRTCGD--------VHGKSKGSVYNIK--TGIW----SDKGSRIC-SC----DNAIA--RRCIKTN---RAAKDKRY-CD-CGKT--SRGN-NVH-TRHKGTK---CK-CDRKTHN------HVRIKHRG---YVCKYCGR--DNCKRHRKHN------DHRYAC-ICNKG---KKKSKTHGVVH-TGH-------CNCAHNRRNS-RTHYKSKHRTKM

>CG17806-vir

LCRTCGQHALNSKHLFDEDGMEIIRNIKILTGILITSQADMPENICVNCLLDVNNAIEFRERCIQMH---RKKPTEPFFCDQCGKSFWEKGNLVVHMKRH-DAKKYECPECGRKEFTAHLLNLHIRIKHKGELPYECKFCGQRFDNSLKRLKHERVKYVFPRPYKCQICDKSFKDKTSFAKHGSIH-SGEKPHQCEVCQTSFNQKRTLVNHYRSKQHIKN

>CG17806-wil

LCRTCGQHALNSKHLFDEDGMEIIRNIKILTGILITSQADMPENICVNCLLDVNNAIEFRERCIQMH---RKKPTEPFFCDQCGKSFWEKGNLVVHMKRH-DAKKYECPECGRKEFTAHLLNLHIRIKHKGELPYECKFCGQRFDNSLKRLKHERVKYVFPRPYKCQICDKSFKDKTSFAKHGSIH-SGEKPHQCEVCQTSFNQKRTLVNHYRSKQHIKN

>CG17806-per-4

PCRTCGEHAVNPRKLFDENGTDILNNLLKLTGIWLTNRTGFPSHICASCLQHSNDAMAFRELCIRTN-KHRYRSIRGFYCDQCGKCFKDKSNLNVHLKRHTGVKQFECEECGRKEFTMHLLSLHMRIKHNGELPYSCKHCGQGFDNCTKRLRHERTECPDTRAHVCHVCRKAFKRKLSLKRHELVH-TGEQPFHCETCHVSFNRKSSLRTHNRSMLHIKK

>CG17806-pse-2

PCRTCGEHAVNPRKLFDENGNDILKDILKLTGVWLTNRTGFPSHICASCLQQTNEAMAFRELCIRTN-KHRYRSIRGFYCDQCGKCFKDKSNLNVHLKRHTGVKQFECEECGRKEFTMHLLSLHMRIKHNGELPYSCKHCGQGFDNCTKRLRHERTECPDTRAHVCHVCRKAFKRKLSLKRHELVH-TGEQPFHCETCHVSFNRKSSLRTHNRSMLHIKK

>CG17806-per-1

LCRTCGEHAVNPRKLFDENGNDILNDILKLTGIWLTNRVGFPSHICASCLQQSNEAMAFRDLCIRTNKKRYRRAIGGFCCDQCGKWFKDKSNLNVHLTRHTGVKQFECEECGRKEFTMHLLSLHIRVKHKGELPYTCKYCGQRFDNCIKRLRHERQECPDIRPHVCHVCGKAFQLKRALRMHEIVH-TGEQPFHCETCDVYFNRKSSLQTHNRSKLHIKK

>CG17806-per-2

LCRTCGEHAVNPRKLFDENGNDILKDILKLTGVWLTNRTGFPSHICASCLQQTNEAMGFRELCIRTN-KKRYRSTRGFYCDQCGKWFKDKCNLNVHLKRHTGVKQFECEECGRKELTMHLLSLHIRVKHKGELPYSCKYCGQRFDNCIKRLNHERRECPDTRPHVCPVCGKAFTRKAELKRHEIVH-TGEQPFHCETCDVYFNQNSSLKTHNRSKLHIKK

>CG17806-per-3

LCRTCGEYAVNPRKLFDENGNDILNDILKLTGIWLTNRTGFPSHICASCLQQTNEAMGFRELCIRTN-RKRYRSTRGYYCDQCGKWFKDKCNLNVHLKRHTGVKQFECEECGRKELTMHLLSLHIRVKHKGELPYTCKYCGQRFDNCIKRLNHERHEFPDIRPHVCPVCGKAFQLKAALRRHEIVH-TGEQPFHCETCDVFFNRKSSLQTHNRSKLHIKK

>CG17806-pse-1

LCRTCGEHSVNPRKLFDENGNDILKDILKLTGVWLTNRTGFPSHICASCLQQTNEAMGFRELCIRTN-KKRYRSTRGFYCDQCGKWFKDKCNLNVHLKRHTGVKQFECEECGRKELTMHLLSLHIRVKHKGELPYTCKYCGQRFDNCIKRLNHERHEFPDIRPHVCPVCGKAFQLKAALRRHEIVH-TGEQPFHCETCDVFFNRKSSLQTHNRSKLHIKK

>Nnk-bia-2

LCRTCGQQAKHFKALFDKEGSDALCNIHKLTGIWFGDHPGVPTKICLSCLLDLNDAVAFRERCIKTNKRKRYKGTKIFICDHCGKKFKDTGNLNMHLMRHTGVKPFECPECGQKELNRYILNIHIRVKHRGEKPYPCKYCGVTFESTTKRVRHVRRKRIK--PFKCSYCDKRFDLKSIRSKHEMVH-TGDRNFPCEICNFSFITKRNRNLHFQTKNHKRK

>CG17806-ana

VCRICNLEAKNFKNLFDNESLEILNNIFKLTGISLTDQDGVPNRICSACSLDLDGAIAFRERCLITN----SLEQGHYICDQCGKTFSEKGNFNVHLTRHMGVKQFQCQECDRSEFTMHLLKLHVRIKHRGELPYVCKYCGQRFKNCNIRLKHERRESPVHRPHLCHICGKAFLDKESLRLHSVVH-TGEQPYHCELCKVKFNRKSSLGTHYRSKIHKKK

>CG17806-kik-2

LCRTCGQGAEYSRDLFDQESTDILGNILKLTGILLTNENGVPTRICVSCLLDLKEAIAFRERCITTNRRIRAMAKGLYFCDQCGKSFNDSSNFNTHLTRHTGVKKFECEECGRKEFTQHLLNLHVRIKHRGELPYVCKYCGQRFGNCNYRLKHERDHNPGPRKYKCPICDKGFKDSKTLKDHGLVH-SGEHPFHCKLCQTHFGKKTSLKTHLRSIGHRKR

>CG17806-kik-1

LCRTCGQEAQHSRTLFDKDATDILFDVLKLTGIWFTDKQGMPTRICMSCQLDLKEAIAFRERCIRLNKRIHAREEGLYFCDQCGKSFSEKGNFNVHLTRHTGLKQFECEECGRKEFTLHLLKLHVRIKHRGELPYVCKYCGQRFDNCIKRLRHERGESPVHRPHTCAICKKAFKDKITLRFHAVVH-TGEQAFHCELCQASFNRKSSLRTHFRSKQHIKR

>CG17806-tak-1

VCRTCGQEAEHAKGLFDKQGSDILGNIFKLTGIRLTDHPGVPTKICLSCLLDLNDAITFRERCIKTNNKGNAVDKKLHFCDQCGKSFAEKSNFNVHMKRHTGTREFQCQECDRKEFTQHLLNLHVRIRHRGELPYVCKYCGQRFNNCLKRLTHERNESPVHRPHVCPICKKAFKNSQTLKNHSVVH-TGEQPFHCELCQTSFNRRNALATHKKSKHHRLK

>CG17806-mel_

LCRTCGQEAEHAKSLFDKEARDVLSNILKLTGFWLRNQPGVPTRICLSCLLDLNDAIAFRERCIRTNKRAYALEHRLYFCDQCGKTFSEKGNFNVHLRRHKGTKEFQCQECDRMEFTQHLLNLHVRIKHRGELPYVCKYCGKRFDNCLKRLNHERNESPVHRPHVCSTCQKAFKTSTALKDHIVVH-TGEQPFHCELCQTFFNRRNALATHYKSKHHRLK

>CG17806-sec_

LCRTCGQEAEHAKALFDKEARDVLSNILKLTGLWLRNLPGVPTRICLSCLLDLNDAIAFRERCIRTNKKAYALEHRLYFCDQCGKTFSEKGNFNVHLRRHKGTKEFQCQECDRMEFTQHLLNLHVRIKHRGELPYVCKYCGKRFDNCLKRLNHERNESPVHRPHVCYTCQKAFKTSTALKDHSVVH-TGEQPFHCELCQTFFNRRNALATHYKSKHHRLK

>CG17806-sim

LCRTCGQEAEHAKALFDKKARDVLSNILKLTGLWLRNLPGVPTRICLSCLLDLNNAIAFRERCIRTNKRAYALEHRLYFCDQCGKTFSEKGNFNVHLRRHKGTKEFQCQECDRMEFTQHLLNLHVRIKHRGELPYVCKYCGKRFDNCLKRLNHERNESPVHRPHVCSTCQKAFKTSTALKDHSVVH-TGEQPFHCELCQTSFNRRNALATHYKSKHHRLK

>CG17806-ere

LCRTCGQEAEHAKALFDQEGSDVLSNILKLTGLWLRNQTGVPTRICLSCLLDLNDAIAFRERCIKTNKRAYALEHRLYFCDQCGKTFSEKGNFNVHLRRHKGTKEFQCKECDRKEFTQHLLNLHVRIKHRGELPYVCKYCGKRFDNCLKRLNHERNESPVHRPHVCSTCQKAFKTSTALKDHSVVH-TGEQPFHCELCKTFFNRRNALATHYKSKHHRMK

>CG17806-yak_

LCRTCGQEAEDAKALFDKEGSDVLNNILKLTGLWLRNQSGVPTRICLSCLLDLNDAIAFRELCIKTNKRAYALEHRLYFCDQCGKTFSEKGNFNVHLRRHKGTKEFQCKECDRKEFTQHLLNLHVRIKHRGELPYVCKYCGKRFDNCLKRLNHERNESPIHRPHVCSTCQKAFKTSTALKDHSVVH-TGEQPFHCELCQTSFNRRNALATHYKSKHHRMK

>CG17806-fic

LCRTCGQEAEHAQSLFDKKSSDVLYNILKLTGLWLSEKPDVPSRICLSCLLDLKEAIAFRERCIKTNQRARAKELRLFFCDQCGKTFAEKCNFNVHLTRHKGTKEFQCQECDRREFTQHLLNLHVRIKHRGELPYVCKYCGQRFDNCLKRLFHERNESPVHRPHVCPVCKKTFKNKQGLKNHSVVH-TGEQPFKCELCQSHFNRRNSLQTHFKSKQHRLR

>CG17806-ele-1

LCRTCGQEAEHGRSLFDKEGSDVQHNILRLTGILLSDEPGVPTMICLSCLLDLNEAIAFRERCIKTNKRAIAKEKKLYFCDQCGKTFSEKGNFNVHLTRHKGTKEFQCKECDRREFTQHLLNLHVRVKHRGELPYVCKFCGQRFDNCLKRLFHERKEDPNHRPYVCSICNKGFKKKEVLKNHSVTH-TGEQPFHCELCQAHFNRRNSLATHFKSKQHQKK

>CG17806-rho-1

LCRTCGQGAEHAKTLFDKEGSDVLYNILKLTGLLLCEKPGLPSRICLSCLLDLNEAIAFRERCIKINKRAHAKEQRLYFCDQCGKTFSEKGNFNVHLTRHKGTKEFQCKECDRKEFTQHLLNLHVRVKHRGELPYVCKFCGQRFDNCLKRLFHERKESPDQRPHVCPICKKAFKTRDVLKKHGVVH-TGEQPFHCELCQTHFNRRNSLVTHFKSKQHQKK

>CG17806-tak-2

MCRTCGQQVDQTQSLFDKDSTEVLYNIFKLTGVLLSDKPGVPTMICLPCVKDLNAAISFRERCIRTNKRAQAKDQKPYFCDQCGKTFSERGNFNVHLTRHKGTKEFQCQECDRREFTQHLLNLHVRVKHRGELPYVCKYCGQRFDNCLKRLFHERKESPDHRPHVCPICKKGFKSTTSLRNHSVVH-TGEQPFHCELCQTHFNRRNSLRTHYKSKQHIIK

>CG17806-bia

LCRTCGHEMDLGKALFHKENSEVLYNILRLTGILFEDHPGVPTEICTSCLLDLTEAIAFRERCIVTNRRAETKDQKLYFCDQCGKTFSEKGNFNVHLTRHKGTKEFQCSECDRREFTQHLLNLHVRIKHRGELPYECKYCGQRFDNCLKRLFHERREHPNQRPHVCPICKKGFKSTTSLKHHNVVH-TGEQPFHCELCQTHFNRRNSLRTHYKSKQHLNK

>CG17806-suz-2

LCRTCGQQAEHSKALFDKEGNDALCNIHKLTGIWFGDHPGVPTKICLSCLLDLNDAVAFRERCIKTNRRAETKDQKLYFCDQCGKTFSEKGNFNVHLTRHKGTKEFQCSECDRREFTQHLLNLHVRIKHRGELPYECKYCGQRFDNCLKRLFHERRENPNQRPHECPICKKGFKSTTSLKHHSVVH-TGEQPFYCDLCQTHFNRRNSLRTHYKSKQHLNK

>CG17806-ele-2

LCRTCCQEAEHAKMLFHKERSYVLHNILKLTGIWLSEKPGVPSRICLTCLLDLHKAMAFRERCIKANQRALAKEQRPYFCDQCGKTFSEQGNFNLHLKRHKGVREFQCKECDRRFFSQHLLSLHVRIKHRGEKPYKCKYCGQRFHNCLRRLDHERNECPDHRPYVCPICNKAFKKNRMLKFHGVVH-TGEQPYQCEICKTHFNRMSSLRTHFKSKQHRLR

>CG17806-rho-2

LCRTCSQDAEHARSLFDMKGSDVLQNILKLTGIWLSDKPGVPSRICLSCLRDLNKAIAFRERCIKTN------KKVEFFCDQCGRSFTEKGNFNLHLKRHLGIREYQCKECDRREISQHLLNLHVRIKHRGEKPYVCKYCGQRFNNCLRRLDHERNESPDHRPYVCHVCNKAFKKKRMLNHHRVVH-TGEQPYHCELCQSHFNRKNSLRTHFKSKHHQSR

>Odj_moj_-1_

CCRTCAEFHLNPRNLFKEENEDIRQNIQSITGIRLSYDPQMPTHICSCCYLDLDHAIAFRERCLDANPKEMSLE-KKYVCEHCGWSFRDLSNMKDHALRHSGVKKFECEECCRKFFTRPLLKLHIRVHHKGEKPFVCKYCGMAFRNSPSRCRHERKYHPNELPFECDICDQTFISKISLDKHKEVHVKGEITHRCETCNKDFKGSTYLRNHYLTKLHQRR

>Odj-gri

HCRTCGEYNLNPRNLFSHENKDIRKNIEAITGIKLSYHSHLPSHVCSCCYLDLNHSMAFRQRCVVTHPR-IAPLEKKFVCDQCGWSFRDLSNMKDHAVRHSGVKNFECEECGCKFFTRPLLMLHVRVHHKGEKPFVCKYCGMAFRNSPSRCRHER---------------------------------------CETCKKTFKGAIFLKNHYTTKFHQRR

>Odj-vir

HCRTCREFNLKPRNIFSHENEDIRHNILAITGIKLFYDPCLPSHICSCCYLDLNHSMAFRERCLESEPR-LPPEEKKYVCDKCGWVFRDLSNLKDHALRHSGVKKFECQDCGSKFFTRPLLLLHIRVHHKGEKPFVCKYCGMGFRNSPSRCRHERKYHANELPFECNLCTRTFISRISLEKHKLVHITGEETYSCKTCNKSFKGAQYLRNHYLTKFHLKR

>CG17801-vir-3

QCRLCAHDEQSAKDIF---NAELVLNVKVLTGILLVQSIDLPRSICSRCILDLNHSIAFRECCIKTNMSRRPKVAASLVCDQCGRSFDNSSNLKLHLVRHTGVKSFECPECDQKFFTNPLLQMHISVRHKGQKPWKCRFCSQGFRTGDARCRHERKEHYN-TKFPCKVCGKSFITKSCLSKHEFLH-TGLRPYRCEICDIGFPRNTQLKIHCKSKSHKKT

>CG17801-wil-2

QCRLCAHDEQSAKDIF---NAELVLNVKVLTGILLVQSIDLPRSICSRCILDLNHSIAFRECCIKTNMSRRPKVAASLVCDQCGRSFDNSSNLKLHLVRHTGVKSFECPECDQKFFTNPLLQMHISVRHKGQKPWKCRFCSQGFRTGDARCRHERKEHYN-TKFPCKVCGKSFITKSCLSKHEFLH-TGLRPYRCEICDIGFPRNTQLKIHCKSKSHKKT

>CG17801-wil-1

YCRLCNEHNRNAPNIF---HIDILKNVAAITGIRLLNRPQIPQHICASCHLDLNHLMVFRERCIKTQKRVPASGVPELVCDKCGKCFKDPSNLKLHLVRHTGVKNFECALCGEKFFSQHLLNLHDRVRHQGERPYKCKFCGLQFLTSTARCRHERIRHIRTLSFKCKYCDKGFIVQSDLKKHEFLH-SGERPHRCEICNIGFPRSTNLKLHFRSKTHQKK

>CG17801-moj-1

KCRTCAVLNPNAKNLF---DNEILNDIETLTGIRLKDDQHMPRHICTCCHLDLYHSIAFRERCLKTENVKPPANPKVFICDLCGHQSSSPKNLDIHILRHKGEKNFECAECGIKHYSKYLLQLHIRVKHQGEMPYLCKFCDQRFYSASTRQRHEQVRHIRSWSYECKICGKKYNTKSCLNKHEFLH-TGLRPYRCDLCNVAFPRKPGLRIHCRTKQHQKR

>CG17801-vir-1

QCRTCAAFNPNAKNLF---DNEILLNIETLTGIRLNEDQHMPKHICTCCHLDLYHSIAFRERCLKTEALKPPGDPKVYICDLCGHQSTSPKNLDIHILRHKGEKNFECEECGAKHYSKYLLQLHIRVKHQGEMPFVCRFCDQRFYSGSTRTRHEQVRHIRSWSYECKICGKKYNTKSCLNKHEFLH-SGLRPYRCELCNVAFPRKPGLRIHCRTKQHQKR

>CG17801-gri

LCRTCGEYHLNATNLF---SREIVENIATLTGIRFDEGEDMPKHICPCCLLDLNYSIAFRERCINVQLRTQSAVIKKFVCDQCGKCFADHSNLKVHILRH-GIKNFECPDCDAKYYTNHLLNLHIRVRHKGEKPYACKYCGQRFFTSTARCRHERVKHTKKYSFVCKYCGKTYLTKSCLNKHEFLH-TGQRPFLCEICNVAFPRKTNLKLHYRSKQHQRR

>CG17801_moj-2_

LCRTCGEYNLNAKNLF---NEDLLHKIDILTGIRLNDEEEMPKHICTCCYLDLNHSIAFRERCIKIQSVKQRGASKSYVCDQCGRCFTDASNLKVHILRHTGVKNFECPECDSKYFTRHLLNLHIRVRHQGEMPYACKYCDQRFFTSTTRCRHERVKHTRKLTYACRLCGKTYLTKSCLNKHEFLH-TGERPYRCDICNVGFPRKTNLKIHYRSKQHQTR

>CG17801-vir-2_

LCRTCGEYNLNAKNLF---SYEMLDKIEILTGIRLDDDEDMPKHICACCYLDLNHSIAFRERCIKIQNARQTGISKTYVCDQCGKCFTDSSNLKVHILRHTGVKNFECEECNTKYFTRHLLNLHIRVRHQGEMPYACKYCDQRFFTSTSRCRHERVKHTRKLTYACRLCGKTYLTKSCLNKHEFLH-TGERPYRCEICNVGFPRNTNLKIHYRSKQHQKR
