# Supplementary material for: Innovation of heterochromatin functions drives rapid evolution of essential ZAD-ZNF genes in Drosophila
Source: eLife. 2020 Nov 10;9:e63368. doi: 10.7554/eLife.63368 (PMC7655104; doi:10.7554/eLife.63368)
Supplement: Supplementary file 4. — Nnk-mel rescue transgene was recoded by synonymous substitutions to make it impervious to RNAi-mediated knockdown, and the Nnk-sim rescue transgene was codon-optimized to match D. melanogaster codon preferences. [file elife-63368-supp4.doc]

>*D. melanogaster* recoded Nnk

ATGACGCCACAATGCCGGTTGTGTGGAGATTTAATATACACGCAAAATCCAGTTAACATATTCGATGAGAACAGCAAAATGGTCAGGCAAATCGCATTGGTCACGGGCCTTTGGGTGAGTGTGTAAATTAAATTGGCAAATTTAAACCGCTAATGCTCGTTTGGTTTTCAAGCTCACCGACCACTCGAAGATGCCCCGGAATATGTGCTCCTGCTGCCTGCTGAGCCTGAAGAGCGCCATTGCCTTCCGGCAAGCCTGCATCAAGACCAACAATCGACTGACAATCCAGCGCCGAAGTGTGGAAGCCAAGGATGACGGATGCTGGGCTGATCCGCTGAGCGATGCGCACGAGGGGCTGAAGATCAACGATGCGGAAGTGGAGCAGGAGGTGTTGTACGAGGTGTCCTATGCGGATAATGAAGGACTGGAGGAAGACCACGACCTGTACAAGAAGGAGGAGGAAGAAAGCGAGGTTAATGAGAACAAGGAATTTGAAGATATTGCCTGCGAAATACCCTTCAGTAAGGAGGATGATGAGCAAGAGCAACAGCAGGAGTATGAGGATATGGTGGATAAAAAGACAGGACATGATGATGGCGAGGAGTCACAGGAATGTGAGGAGTCACAGCCAGATGAGGAGGAAAGTCAGCAAAATGAGGAGGACGAAGAGGAGTCACAGGAAGACGACGACGAGCTTTGGCAGAACGAGGACGGCGACAGCGATACAGATGCTGATTCTATGTCAGATATAGAAGCCACATCGCGTCAATCGGCCCTTGACGAAGATAAGAAACCGCGCCGCAAGTATACGAAACGCTCCTCCCCGAAGAATGATGATACTGATTTCCTGAAGCCGGCAAAAAAGAAGCGGAAGACCTATATCTCCCAGAAAGTGCATATTTGCGATCACTGTGGAAAAAAGTTTACGGATAAAGGAAATTTCAACTTGCATGTATTGCGTCATTCCGGAGTTAAACCATTTGAATGCCCCGAATGTGGCCAGAAAGAATTTAATCGCTATATTCTCAATATCCATATCCGTGTTAAACATCGTGGTGAAAAACCCTATGCGTGCCAGTTCTGCGACGAGCGTTTTGTGCACAGTACTATGCGATCGCGCCACGAAAAGTGAGTCTATAGCATACCTTAAATCCCCTAAATTGCACCTACGAACACTGCCATTCTTTCAGCCGGGTACACCGGAACAAGAAAACACCAAAGAACTTCAAGTGCAACTATTGCGACAAGCGATACGAGTCGAACTACCAGAGGGCCAAGCACGAGGTGGTCCACACCGGCGAGCGTAACTTCCAGTAAGATCTTGTGAAGATATATGGCTAGATTTTTCTTTCTCCTAACATATTCCTTTAAGTATCAATCATCTTATTATTCCCACTTTGTAACTCTTTACTTCCTAATATCCACTACATCCAGCAATCAAGTTAACTATTTCCTGCTTGAAAAGTTACTTTTTCAAATGTTTAACTTTGTTTTTATCTACTTTCCCCTTAGCTGTGAGGTCTGCAAAGTGTCCTTTACACGGAACTCGAACTTGAAGACCCACTACCGCTCCAGGCAGCATCAAAATAAGCTGATCGCAATGAGTGCTAAGTCCGAAATTGATTCCAAAAATAAATAA

>*D. simulans* recoded Nnk

ATGGCTCGAAAAATAACTCTTAAAAATGACGCCCCAGTGCCGCCTGTGCGGAGATAGCATTTATACCCAGAATCCTTTTAACATTTTTGACGAACGATCCAATATGGTCCGTCAGATCGCCTTGGTAACTGGTTTGTGGTTGACAGACCATTCCAAGATGCCAAGGAACATGTGTTCCTGCTGTCTCCTGTCGTTGAAAAGTGCTATTGCATTCAGGCAGGTGTGTATAAACACGAACAACCGACTCACTATACAGCGGCGGCGGCTCGAGGCAAAGGATGACGGCTGTTGGGATGATCCTCTCAGTGATGCACATGAGGTTTTGAAAATCAATGATGCGGAGGTAGAGCAAGAAGTGCTCTATGAGGTTAGTTATGCAGACAATGAAGGCCTGGAGGAGGATCACGACTTGTTCAAGAAAGAGGAGGAAGAATCCGAAGTTGACGAACAGAAAGAGTTCGTCGACACTGCCTCCGAAATACCAAGCAATAAGGAGGACAATGATCAAGAGCAAAAACAAGAGTGTGAAGATATGGTTGATAAGAGGAAAGGTCATGACGCCGGTGAAGAGTCCCAGGAGTACGAGGAGTCGCACCCAGACGAAGAAGATGAAGAGGAGTCGCAAGAAGAGGACGATGAACTCTGGCAAAACGAAGACGGCGACAGTGACACGGACATTGCGTCCATGTCGGATCGGGAGGCAACGTCGAGGCAACCGGCTTTGGAGGAGGATAAAAAGTCCATCCGGAAATATACTAACCGAAGTAGTCCTAAAAACGACGATACGGACATCCTCAAGCCTGCAAAAAAAAAACGAAAGACATATATTAGTCGTAAAGTTCACGTCTGTGATCATTGCGGCAAAAAGTTCACTGATAAAGGAAACTTCAATCTGCACGTTTTGCGACATAGTGGTGTGAAACCTTTTGAGTGCCCAGAGTGTGGACAGAAAGAGTTCAATAGGTATATACTGAACATACATATACGAGTTAAGCATAGGGGTGAGAAACCATATGCGTGTCAATTCTGTGACGAGAAGTTCGTGCACAGTACGATGAGGAGTAGGCACGAAAATAGGGTGCACAGGAACCGTAAGTCCCCTAAGAACTTTAAATGCAATTACTGTGACAAAAGGTTTGAATCGAATTATCAACGTGCAAAACACGAGGTAGTGCATACAGGAGAACGCAAATTTCATTGTGAGGTATGTAAGGTCAGCTTTACGCGCAACTCCAATTTGAGGACGCACTACAGGAGCGGACAGCATCAGCGGCGACAAATTGCCATGTCGGCCAAGTCCGAGAAAGACAGCAAGAACAAGTAA
